# Extracorporeal shockwave therapy in bone fractures: a systematic review

**DOI:** 10.1007/s00402-026-06349-6

**Published:** 2026-05-24

**Authors:** Thatiane Izabele Ribeiro Santos, Lais Caroline Souza e Silva, Mirian Bonifacio, Cassia Maria Campos Melo, Bruna Genari Sena, Mariana Carvalho Simões, Julia Risso Parisi, Ana Caroline da Silva Fernandes, Helena Mayumi Akashi, Flavia de Oliveira, Ana Claudia Muniz Renno

**Affiliations:** 1 Federal University of São Paulo, São Paulo, Brazil; 2 Universidade Metropolitana de Santos, Santos, Brazil

**Keywords:** Extracorporeal shockwave therapy, Bone fractures, Fracture healing, Nonunion

## Abstract

**Introduction:**

The increasing incidence of fractures and cases of delayed union or nonunion has encouraged the search for non-invasive therapies. Extracorporeal shockwave therapy (ESWT) has been proposed as a strategy to stimulate bone regeneration and improve fracture healing.

**Materials and methods:**

A systematic review was conducted following PRISMA guidelines, with searches performed in PubMed, Scopus, Web of Science, and Embase (2005–2025). Clinical studies evaluating ESWT for fracture treatment with radiographic assessment of bone healing and the presence of a control group were included.

**Results:**

A total of 834 studies were initially identified, of which 7 were included in the final analysis. Most studies demonstrated positive effects of ESWT, including improved bone healing, pain reduction, and functional recovery. However, there was considerable heterogeneity in treatment protocols and relatively small sample sizes.

**Conclusion:**

ESWT shows potential as a non-invasive therapy to stimulate fracture healing, particularly in cases of delayed union or nonunion. However, further controlled clinical trials and standardized protocols are needed to confirm its clinical effectiveness.

## Introduction

In recent decades, the incidence of fractures has increased significantly worldwide, mainly due to the marked rise in accidents and chronic degenerative diseases. Moreover, fractures represent a significant and widespread public health challenge characterized by high morbidity, mortality, and substantial costs depending on the extension of the fracture [1]. It is well known that bone repair following a fracture is known to be a highly complex process that involves the interaction of multiple biological events, including active gene expression and the coordinated action of numerous cells and proteins, ultimately leading to restoration of bone tissue integrity [2] Despite this complexity, in most cases bone tissue is capable of regenerating itself and undergoing self-repair, culminating in complete fracture healing and restoration of tissue function [3, 4].

However, in certain situations, such as in the presence of diseases (e.g., osteoporosis) or large bone defects, this process may be impaired and the regenerative response insufficient, resulting in delayed union or nonunion. In the United States alone, the total number of bone fractures is estimated to exceed 15 million annually, of which 10–15% may lead to complications and/or delayed healing [5]. Consequently, the treatment of such fractures is extremely challenging and typically relies on surgical interventions involving prosthetic devices and metallic fixation systems. Nevertheless, due to poor bone quality, osteointegration with the implanted material often fails to occur, leading to persistence of the clinical problem [6].

In recent years, the therapeutic effects of osteogenic resources aimed at improving both the quality and quantity of bone tissue, as well as enhancing osteointegration between bone and metallic implants, have shown increasingly promising results [7, 8]. One of the most promising resources is the extracorporeal shock wave therapy (ESWT) [9]. ESWT is a non-invasive therapeutic procedure in which a single-impulse transient acoustic wave of 1 microsecond duration is applied to different target body regions to produce analgesia and facilitate healing through a mechanism called mechanotransduction [10]. Shock waves are considered an effective, non-invasive and cost- and time-efficient treatment [10, 11] The physiological mechanisms of its therapeutic effect are based mainly in the mechanotransduction in its passage through the tissues, achieving an analgesic, osteogenic, neovascular and tissue repair effect [12]. Among the beneficial effects they produce, it is worth highlighting that they produce analgesia, facilitate protein synthesis, increase vascularization, improve cell proliferation, produce calcium destruction in tissue and have a protective effect on cartilage and bone [12].

More specifically, the bio-stimulatory effect on osteogenesis has been demonstrated to increase bone repair and regeneration by triggering the release of transcription factors, mediators, and growth factors [13]. Due to these positive effects, shock waves are thought to have promising results in the treatment of fracture healing, osteonecrosis, osteoarthritis, and osteoporosis [14]. Until now, studies of the effect of ESWT on osteoporotic bone tissue healing have been limited, and the focus of these has been to accelerate fracture healing [15].

Inoue et al. [16], in a study using an in vivo model of femoral bone defects and treated with shock waves, demonstrated an accelerating of fracture healing. Similarly, Koolen et al. [17] investigated the effects of extra corporeal shockwaves on the process of osteointegration in femur screw fixation in a model bone defect in rats. As a result, authors observed the formation of neocortices, acceleration of bone formation and improved screw fixation after the treatment.

Despite the positive effects of extracorporeal on the process of bone healing, there is still a limited understanding of their biological interaction and the process of bone tissue stimulation. In this context, the purpose of this study was to review the literature investigating the effects of different materials on the process of bone healing in clinical trials.

## Materials and methods

### Review protocol

To conduct this systematic review, four databases were searched: PubMed, Scopus, Web of Science, and Embase, covering the period from 2005 to 2025. The literature search was performed in accordance with the Preferred Reporting Items for Systematic Reviews and Meta-Analyses (PRISMA) guidelines. The review protocol was prospectively registered in the International Prospective Register of Systematic Reviews (PROSPERO) under registration number CRD420251246364. Initially, Medical Subject Headings (MeSH) terms were identified and combined using Boolean operators, as shown in Table 1. In addition, synonyms were searched in the title and abstract for each component to ensure comprehensive retrieval of relevant studies.

**Table 1:** Search strategy containing all terms and synonyms retrieved in the databases

Terms	Search
Component 1: Shockwave	(“Extracorporeal Shockwave“[MeSH Terms] OR ESWT OR “shockwave” OR “extracorporeal shockwave” OR “ESWT“[All Fields] OR “shockwave“[All Fields] OR “extracorporeal shockwave“[All Fields])
Componente 2: Fractures	(“Nonunions“[All Fields] OR “Delayed unions“[All Fields] OR “Acute fractures“[All Fields] OR (“injuries“[MeSH Subheading] OR “injuries“[All Fields] OR “trauma“[All Fields] OR “wounds and injuries“[MeSH Terms] OR (“wounds“[All Fields] AND “injuries“[All Fields]) OR “wounds and injuries“[All Fields] OR “trauma s“[All Fields] OR “traumas“[All Fields]) OR (“fractur“[All Fields] OR “fractural“[All Fields] OR “fracture s“[All Fields] OR “fractures bone“[MeSH Terms] OR (“fractures“[All Fields] AND “bone“[All Fields]) OR “bone fractures“[All Fields] OR “fracture“[All Fields] OR “fractured“[All Fields] OR “fractures“[All Fields] OR “fracturing“[All Fields]) OR “Bone fracture“[All Fields] OR “fractures bone“[MeSH Terms] OR “Pseudarthrosis“[Title/Abstract] OR “Osteoporosis“[All Fields] OR “Osteopenia“[All Fields] OR “Fracture-Dislocation“[Title/Abstract] OR “Stress Fracture“[Title/Abstract] OR “Bone Stress“[Title/Abstract] OR “Osteotomy“[Title/Abstract])
Component 3: Bone Healing	(“Consolidation“[Title/Abstract] OR “Bone healing“[Title/Abstract] OR “Fracture healing“[Title/Abstract] OR (“healed“[All Fields] OR “wound healing“[MeSH Terms] OR (“wound“[All Fields] AND “healing“[All Fields]) OR “wound healing“[All Fields] OR “healing“[All Fields] OR “healings“[All Fields] OR “heals“[All Fields]))

### Study selection

Based on the predefined inclusion and exclusion criteria, potentially eligible studies were identified. The literature search and initial screening were independently performed by two reviewers (CMCM and TISR), who assessed the titles and abstracts of the retrieved records. Subsequently, three reviewers (CMCM, TISR, and BGS) evaluated the full texts of the selected studies and reached a consensus regarding their eligibility. Articles that failed to meet the established criteria were excluded during the full-text review stage.

### Eligibility criteria inclusion criteria

(i) Clinical studies.
(ii) Studies investigating the use of extracorporeal shock waves for fracture treatment.
(iii) Studies assessing bone healing or fracture consolidation as an outcome.
(iv) Studies including a control group.
(v) Studies evaluating bone consolidation by radiographic analysis.
(vi) Articles published in the English language.
(vii) Studies published between 2005 and 2025.

### Exclusion criteria

(i) In vitro, ex vivo, in vivo (animal), and review studies.
(ii) Studies that did not involve shockwave therapy applied to fractures.
(iii) Studies without outcomes related to bone consolidation or healing.
(iv) Studies without a control group.
(v) Studies that did not use radiographic methods to assess bone healing.
(vi) Articles published in languages other than English.
(vii) Studies published outside the defined time frame.

### Data extraction

For this systematic review, the primary outcome assessed was bone healing, defined by radiographic evidence of fracture consolidation following extracorporeal shockwave therapy. In addition, secondary data were extracted from the included studies, comprising authorship, year of publication, study design, fracture type, anatomical location of the fracture, shockwave treatment parameters, presence and characteristics of the control group, follow-up period, imaging methods used for bone healing assessment, and the main outcomes related to fracture consolidation.

### Types of reported results

To assess the risk of bias of the included studies, the Risk Of Bias In Non-randomized Studies of Interventions (ROBINS-I) tool was applied [18]. This instrument is specifically designed to evaluate the risk of bias in non-randomized studies that aim to estimate the effects of interventions. The ROBINS-I assesses bias across seven domains: bias due to confounding, bias in selection of participants into the study, bias in classification of interventions, bias due to deviations from intended interventions, bias due to missing data, bias in measurement of outcomes, and bias in selection of the reported result. Each domain was rated as low, moderate, serious, or critical risk of bias, and an overall risk of bias judgment was assigned for each study according to the highest risk identified across domains. In addition, the GRADE (Grading of Recommendations Assessment, Development and Evaluation) approach was used to assess the certainty of the evidence for the main outcomes [19]. The certainty of evidence was evaluated considering the study design, limitations, inconsistency, indirectness, imprecision, and publication bias, and was classified as high, moderate, low, or very low. The GRADE assessment was applied at the outcome level, providing an overall judgment of the certainty of evidence for fracture union rate and time to fracture union.

## Results

**Fig. 1:**
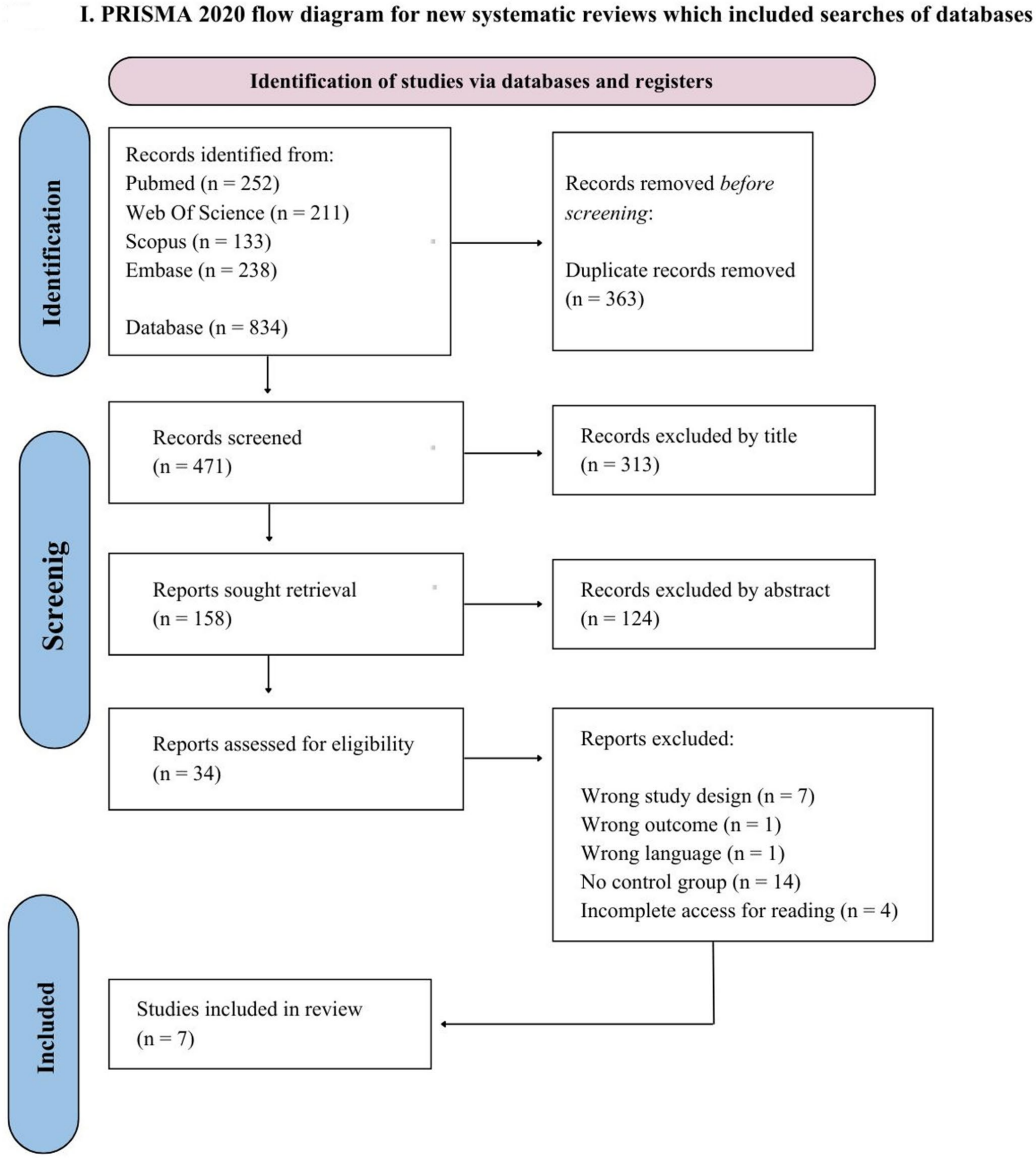
PRISMA 2020 flow diagram for new systematic reviews which included searches of databases

The flow diagram outlines the search approach adopted in this study. Figure 1 illustrates how articles were screened and filtered through the inclusion and exclusion phases. A total of 834 studies were initially identified in the databases PubMed (252), Web of Science (211), Scopus (133), and Embase (238). After removing duplicates (*n* = 363), 471 records remained for assessment. From these, 313 articles were excluded during the title review, leaving 158 for further analysis. Screening of the abstracts removed an additional 124 studies, resulting in 34 papers selected for full-text evaluation. Among these, 7 were removed for having unsuitable study designs, 1 due to irrelevant outcomes, 1 because it was not written in English, 14 for lacking a control group, and 4 because full access was unavailable for reading. A final sample of 7 studies met the eligibility criteria and were included in the review.

The general characteristics of the studies included in this review are summarized in Table 2, detailing author and year of publication, country, study design, sample size, mean age, sex distribution, type of fracture, anatomical site, and follow-up period. The included studies were published between 2007 and 2025, comprising investigations conducted by Ahmed et al. [20], Gökalp et al. [21], Mittermayr et al. [11], Quadlbauer et al. [22], Ramon et al. [23], Wang et al. [24], and Yang et al. [25].

These studies were carried out in different countries, including Egypt [20], Turkey [21], Austria [11], several European centers [22], Spain [23], and China [24, 25], demonstrating a broad geographical distribution. In terms of study design, one randomized controlled clinical trial was identified [20], along with prospective randomized clinical trials [23], prospective open clinical studies [11], retrospective evaluations [21, 24, 25], and retrospective follow-up studies [22]. The duration of data collection varied widely, ranging from short-term retrospective analyses to long-term prospective cohorts spanning several years [11].

Sample size varied across studies, with a total number of participants ranging from 18 to 68 patients. pecifically, Ahmed et al. [20], included 21 participants equally distributed into three groups, Gökalp et al. [21] evaluated 68 patients divided between decortication and ESWT groups, and Mittermayr et al. [11] analyzed 49 patients allocated to ESWT and surgical treatment groups. Quadlbauer et al. [22] included 42 patients, Ramon et al. [23] evaluated 18 patients equally distributed between ESWT and surgical groups, Wang et al. [24] included 56 patients divided into two equal groups, and Yang et al. [25] assessed 68 patients distributed among control, ESWT, and ESWT combined with kinesiotaping groups.

The mean age of participants ranged from 28.5 ± 5.5 years in patients with fresh mandibular fractures [20] to approximately 57.5 years in patients with tibial and fibular nonunion or delayed union [25]. Studies involving long bone nonunion and delayed union generally included middle-aged adults, with mean ages of 44.1 years [21], 44 ± 13 years in the ESWT group and 43 ± 12 years in the surgical group [11], and 34.2 ± 16.7 years in the Chinese cohort [24]. Younger populations were reported in studies evaluating scaphoid and metatarsal nonunion, with mean ages of 32 years [22] and 21–22 years [23], respectively.

Sex distribution across the studies showed a predominance of male participants. Ahmed et al. [20] reported 12 male and 9 female patients, while Gökalp et al. [21] included 54 males and 14 females. Mittermayr et al. [11] reported 33 male and 16 female participants, Quadlbauer et al. [22] included 40 males and 2 females, and Wang et al. [24] reported 40 males and 16 females. Ramon et al. [23] exclusively evaluated male patients, whereas Yang et al. [25] included 45 males and 22 females.

The included studies addressed a range of fracture conditions across distinct anatomical regions, varying from early-stage injuries to advanced impairments of bone consolidation. Acute fractures were investigated exclusively in patients with recent mandibular fractures, representing an early healing scenario without prior evidence of consolidation failure [20]. Overall, the studies encompassed different stages of the bone healing process, including acute fractures, stress fractures, nonunion, and delayed union, affecting both long and small bones. Nonunion of long-bone diaphysis was reported in tibial and femoral fractures [21], whereas small-bone involvement included scaphoid nonunion [22] and metatarsal nonunion [23]. These investigations focused on fractures characterized by complete arrest of the healing process within their respective anatomical sites. In addition, several studies encompassed cases classified as either nonunion or delayed union, thereby including fractures with varying degrees of biological compromise within the same skeletal segment. This combined pattern was observed in clavicular fractures [11], femoral and tibial diaphyseal fractures [24], and tibial and fibular fractures [25], reflecting heterogeneous consolidation statuses across different long bones.

Finally, the follow-up period ranged from 6 months to 33 months. A 6-month follow-up was reported by Ahmed et al. [20] and Yang et al. [25], whereas most studies adopted a 12-month follow-up period Mittermayr et al. [11], Gökalp et al. [21], Quadlbauer et al. [22]. Ramon et al. [23] reported the longest follow-up, extending to 33 months, and Wang et al. [24] evaluated outcomes at multiple time points, including 1, 3, 6, and 12 months.

**Table 2:** General characteristics of the included studies considering study design, participants, and fracture characteristics

Author (Year)	Country	Study design	Sample size (*n*)	Mean age (years)	Sex (M/F)	Type of fracture/ Anatomical site	Follow-up period
Ahmed et al. 2022 [20]	Egypt	Randomized controlled clinical trial	21 total (*n* = 7 per group)	Mean 28.5 ± 5.5 (range 20–40)	12/9	Fresh fractures/Mandible	6 months
Gökalp et al. 2023 [21]	Turkey	Retrospective evaluation (2007–2015)	68 total (Decortication: *n* = 32 and ESWT: *n* = 36)	Mean 44.1 years (range 24–69) (Decortication: 44.1 (24–69) and ESWT: 43.8 (21–62))	54/14	Nonunion/Tibial and femoral diaphysis	12 months
Mittermayr et al. 2022 [11]	Austria	Prospective open clinical study (1999–2018)	49 total (ESWT group: *n* = 28 and Surgery Group: *n* = 21)	ESWT 44 ± 13 (15–75) Surgery 43 ± 12 (21–62)	33/16	Nonunion and delayed union/Clavicle	6 to 12 months.
Quadlbauer et al. 2019 [22]	Europe	Retrospective follow-up study (2002–2014)	42 total (ESWT group: *n* = 26 and Without ESWT group: *n* = 16)	Mean 32 (range 18–71)	40/2	Nonunion/Scaphoid	12 months
Ramon et al. 2023 [23]	Spain	Prospective randomized clinical trial (2017–2019)	18 total (*n* = 9 per group)	ESWT group: 22 Surgery group: 21	18/0	Nonunion/Metatarsal	33 months
Wang et al. 2007 [24]	China	Retrospective evaluation (January-October 2004)	56 total (*n* = 28 per group)	Mean 34.2 ± 16.7 (range 15–81)	40/16	Nonunion and delayed union/Femoral and tibial diaphysis	1, 3, 6 and 12 months
Yang et al. 2025 [25]	China	Retrospective evaluation (2021–2023)	68 total (Control group: *n* = 28, ESWT group: *n* = 24 and ESWT + KT group: *n* = 16)	Control Group: 55.5 ESWT Group: 57.5 ESWT + KT Group: 53	45/22	Nonunion and delayed union/Tibia and fibula	6 months

*ESWT* Extracorporeal Shockwave Therapy, *KT * Kinesiotherapy, *M* Male, *F* Female

The characteristics of the ESWT protocols applied in the included studies are presented in Table 3, along with their outcomes related to bone consolidation.

Related to the parameters, the energy flux density used across the studies ranged from 0.21 mJ/mm^2^ to 0.62 mJ/mm². The lowest value was reported by Ramon et al. [23], whereas intermediate values were observed in Ahmed et al. [20], Mitternayr et al. [11], and Quadbauer et al. [22], ranging approximately from 0.35 to 0.41 mJ/mm^2^. In contrast, the highest intensities were described by Gokalp et al. [21] and Wang et al. [24], reaching values close to 0.6–0.62 mJ/mm^2^. Alternatively, Yang et al. [25] used parameters based on pressure (2.0–3.0 bar), combined with a frequency of 10 Hz, rather than expressing the dose in terms of energy flux density.

Among the analyzed studies, 3 adopted 3000 pulses per session Mitternayr et al. [11], Gokalp et al. [21], Quadbauer et al. [22]. Ramon et al. [23] applied 2000 pulses per session, whereas Ahmed et al. [20] used 4000 pulses in a single application. Wang et al. [24] applied a higher number of impulses, with 6000 pulses in a single session. Yang et al. [25] reported the application of 2000–3000 pulses per session.

Regarding the number of treatment sessions, five studies reported a single-session protocol Mitternayr et al. [11], Ahmed et al. [20], Gokalp et al. [21], Quadbauer et al. [22], Wang et al. [24]. In contrast, Ramon et al. [23] adopted a protocol consisting of three sessions with a one-week interval between applications, while Yang et al. [25] implemented a longer treatment program comprising 32 sessions distributed over four cycles, with each cycle including two sessions per week.

Regarding the experimental design, the selected studies presented different approaches to participant allocation and the interventions analyzed. Two studies allocated participants to surgery-only and ESWT groups protocol Mitternayr et al. [11], Ramon et al. [23]. Similarly, two studies distributed participants into surgery-only and surgery plus ESWT groups Quadbauer et al. [22], Wang et al. [24]. Ahmed et al. [20] divided participants into three groups: control, ESWT, and low-intensity pulsed ultrasound (LIPUS). Gokalp et al. [21] allocated participants to a decortication and bone grafting group and an ESWT group. Yang et al. [25] further incorporated kinesiotherapy, dividing participants into a control group, an ESWT group, and an ESWT plus kinesiotherapy group.

Conventional radiography was used in two studies Ahmed et al. [20], Wang et al. [24]. In the study by Wang et al. [24], radiographic confirmation of the depth and plane of application was also performed. Mitternayr et al. [11] used X-ray fluoroscopy to precisely place the applicator on failed union, whereas Quadbauer et al. [22] employed an image intensifier to locate the scaphoid nonunion site. Ramon et al. [21] opted for ultrasonography with a linear probe to identify the fracture focus, while Gokalp et al. [21] performed prior radiographic identification of the nonunion site before ESWT application. ESWT was performed under regional or general anesthesia in most studies Ahmed et al. [20], Gokalp et al. [21], Quadbauer et al. [22], Wang et al. [24]. However, Ramon et al. [23] reported the use of sedation. In the protocol described by Yang et al. [25], patients were treated while awake, positioned comfortably, using a coupling agent, and with precautions taken to avoid interference with shockwave transmission.

**Table 3:** Characteristics of extracorporeal shockwave therapy protocols and bone healing outcomes

Author (Year)	ESWT type	Energy flux density (mJ/mm²)	Number of pulses	Number of sessions	Interval between sessions	Groups	Application guidance
Ahmed et al. 2022 [20]	Focused	0.35 mJ/mm²	4000	1 session	N.D.	1. Control group 2. LIPUS group 3. ESWT group	On the day following the surgical procedure, ESWT was applied under regional anesthesia.
Gokalp et al. 2023 [21]	Focused	0.6 mJ/mm²	3000	1 session	ND	1. Decortication and bone grafting group 2. ESWT group	All patients underwent general or regional anesthesia, with radiographic identification of non-union and site marking. ESWT was applied to 3–4 foci in a single session, preserving adjacent anatomical structures. Cryotherapy was applied to the procedure site within the first 24 h.
Mitternayr et al. 2022 [11]	Focused	0.4 mJ/mm² and a frequency of 4 Hz	3000	1 session	ND	1. Surgery group 2. ESWT group	Patients were anesthetized and positioned supine. X-ray fluoroscopy identified and marked the nonunion site. Radiographic guidance was used to position the shockwave therapy head and avoid lung involvement.
Quadbauer et al. 2019 [22]	Focused	0.41 mJ/mm²	3000	1 session	ND	1. Surgery group 2. Surgery + ESWT group	ESWT was performed under general or regional anesthesia. Scaphoid nonunion was localized using an image intensifier. Shockwaves were focused on the nonunion site and applied at three points in the extensor region and across the proximal pole of the scaphoid.
Ramon et al. 2023 [23]	Focused	0.21 mJ/mm² and a frequency of 4 Hz	2000	3 sessions	It was applied once per week for three weeks.	1. Surgery group 2. ESWT group	ESWT was performed under sedation, with the aid of ultrasonography using a linear probe to locate the fracture site.
Wang et al. 2007 [24]	Focused	0.62 mJ/mm² and a 28 kV	6000	1 session	ND	1. Surgery group 2. Surgery + ESWT group	Shockwaves were applied with patients positioned on the traction table, with radiographic confirmation of the treatment site and depth.
Yang et al. 2025 [25]	Focused	2.0 to 3.0 bar, and a frequency of 10 Hz	2000–3000	32 sessions	Over a total of four cycles, patients received ESWT twice per week, with a 3–4-day interval between sessions, and eight sessions constituted one treatment cycle.	1. Control group 2. ESWT + KT group 3. ESWT group	Patients were treated while conscious and positioned comfortably. The fracture site was identified radiographically and prepared in advance.

*MJ/mm²* Millijoule per meter, *kV* Quilovolt, *ESWT* Extracorporeal Shockwave Therapy, *KT* Kinesiotherapy, *ND* Not Determined, *LIPUS*  low intensity pulsed ultrasound

Table 4 presents the imaging methods used to evaluate bone healing after ESWT, the associated outcomes, and the results of each analysis. Different radiological approaches were employed among the included studies. Ahmed et al. [20] used cone beam computed tomography (CBCT) to measure bone density, while Gokalp et al. [21] and Ramon et al. [23] used conventional radiographs in two incidences to assess healing. Mittermayr et al. [11] combined conventional radiographs with computed tomography, Quadlbauer et al. [22] used computed tomography (CT), and Wang et al. [24] and Yang et al. [25] used serial radiographs.

Ahmed et al. [20] observed higher bone density in the ESWT group compared to LIPUS and the control group, although there was no statistically significant difference in the percentage variation. Gokalp et al. [21] identified a significant difference in consolidation rates between the groups, with debridement associated with bone grafting being superior to ESWT. Mittermayr et al. [11] demonstrated similar consolidation rates between ESWT and surgery at three and six months. Quadlbauer et al. [22] reported consolidation in approximately 79% of cases, with no significant difference between ESWT associated with surgery and surgery alone, although techniques with greater stability tended to have better results. Ramon et al. [23] demonstrated complete consolidation and absence of pseudoarthrosis at the end of follow-up. Wang et al. [24]demonstrated earlier consolidation and a greater number of patients with complete healing in the ESWT group. Yang et al. [25] demonstrated a significant improvement in radiographic scores for bone repair, especially when ESWT was combined with kinesiotherapy.

In addition to image analyses, several clinical outcomes, including pain measured by visual analog or numerical scales Ahmed et al. [20], Quadlbauer et al. [22], Ramon et al. [23], Wang et al. [24] and Yang et al. [25]. Quadlbauer et al. [22] analyzed range of motion, grip strength, and fist scores. Ramon et al. [23] evaluated the time required to return to sport activity levels, Wang et al. [24] investigated the function and the return to usual activities and Yang et al. [25] measured walking ability, and Mittermayr et al. [11] performed computer simulation for safety analysis. As a result, Ahmed et al. [20] reported a reduction in pain at the fracture site after one week in all groups evaluated, although patients undergoing ESWT required local anesthesia due to pain associated with the application of high-energy shock waves. Mitternayr et al. [11], using three-dimensional computer simulation, demonstrated that ESWT produced a maximum positive pressure of 23.95 MPa in bone tissue and a maximum negative pressure of − 0.68 MPa, with rapid spatial dissipation of energy, indicating no significant risk to adjacent tissues, such as lung tissue, considering application in the clavicle region. Quadbauer et al. [22] observed substantial functional recovery, with approximately 88% of the range of motion for wrist flexion-extension, 96% for pronation-supination, and about 84% of hand grip strength compared to the uninjured side; furthermore, patients treated with ESWT reported lower pain intensity on the VAS scale and higher scores on the Mayo score. Ramon et al. [23] reported complete pain relief in both groups by approximately three months, progressive improvement in functional scores until full recovery, absence of residual pain or new injuries at long-term follow-up and return to sports with a median of 16 weeks in the surgical group and 13 weeks in the ESWT group, with no statistically significant differences. Wang et al. [24] observed a significant reduction in pain following ESWT treatment, progressive improvement in the function of the affected limb, and a faster return to normal activities compared with the other groups. Yang et al. [25] reported a significant reduction in pain in all groups following the intervention, although there were no statistically significant differences between them, and a clear improvement in walking ability, with a significant increase in FAC scores and greater functional gains in the groups that received ESWT.

The outcomes were classified as positive in most studies, including Ahmed et al. [20], Mittermayr et al. [11], Quadlbauer et al. [22], Ramon et al. [23], Wang et al. [24], and Yang et al. [25], while Gokalp et al. [21] reported negative results for ESWT when compared to debridement with bone grafting.

**Table 4:** Imaging methods and bone healing outcomes following ESWT treatment

Author (Year)	Imaging Method	Imaging Results	Functional Assessment	Results of Functional Assessment	Outcome (+/−)
Ahmed et al. 2022 [20]	CBCT	Bone density was higher in ESWT than in LIPUS and Control. Although the mean density of ESWT was higher, the percentage change in bone density was not statistically significant when comparing the three groups.	VAS	After one week, pain in the fracture area had decreased in the ESWT Group, the LIPUS Group, and the Control Group. There was a difference in the need for anesthesia among the groups, with the ESWT Group requiring local anesthesia due to pain associated with high-energy ESWT therapy.	+
Gokalp et al. 2023 [21]	Two-view radiographs	The debridement and bone graft group showed a significantly higher success rate than ESWT.	ND	ND	-
Mitternayr et al. 2022 [11]	Conventional radiographs and CT	After three months, consolidation occurred in 46% of patients treated with ESWT and in 43% of those who underwent surgery. After six months, consolidation reached 75% in the shock wave group and 71% in the surgical group, with no statistically significant difference at either time point.	Comprehensive three-dimensional computer simulation	ESWT showed a peak positive pressure of 23.95 MPa in the bone and a maximum negative pressure of − 0.68 MPa, with rapid spatial dissipation, showing no significant risk to lung tissue, since it was applied to the clavicle area.	+
Quadbauer et al. 2019 [22]	CT	Consolidation was observed in 81% of ESWT cases and in 75% of those treated surgically, without statistical significance. Regarding stabilization, consolidation occurred in 60% of patients with a headless compression screw, 83% with two screws, and 85% with a plate, without a statistically difference.	Range of motion of the wrist, hand grip strength, VAS, DASH, PRWE, MHQ, and Mayo	Patients recovered approximately 88% of flexion-extension range of motion, 96% of pronation-supination, and approximately 84% of grip strength compared to the uninjured side. The ESWT-treated group had significantly less pain on the visual analog scale and higher Mayo scores, with no statistically significant increase in the rate of consolidation.	+
Ramon et al. 2023 [23]	Conventional radiographs	In 33 months, all patients progressed to bone healing. The median time to healing was 3 months in both groups, with a probability of non-healing of 44% in the surgical group and 33% in the ESWT group, decreasing to 22.2% at four months and reaching 0% at five months.	VAS, time to return to sport, current activity level.	Complete pain reduction was observed in both groups up to approximately three months, progressive improvement in functional scores until full recovery, absence of residual pain or re-injury in late follow-up, and return to sport with a median of 16 weeks in the surgical group and 13 weeks in the ESWT group, with no statistical differences.	+
Wang et al. 2007 [24]	Conventional radiographs and 3D- CT	Compared to the control and conventional treatment groups, the ESWT group showed early consolidation and a higher proportion of complete healing.	VAS, function, and return to normal activities.	ESWT showed a significant reduction in pain after treatment, progressive improvement in the function of the affected limb, and a faster return to activities when compared to the other groups.	+
Yang et al. 2025 [25]	Conventional radiographs	The ESWT + KT group showed significantly greater bone repair, as well as increased muscle mass in the affected limb. Lane-Sandhu radiographic scores increased from mean values between 4 and 6 to approximately 6 to 8 after treatment with ESWT + KT, with a significant difference compared to the control group.	NRS, FAC, Hoffer’s walking ability grade	A significant reduction in pain was observed in all groups after the intervention, although there was no statistical difference between them. Walking ability showed clear functional improvement, with a significant increase in FAC levels in all groups and greater gains in the groups that received ESWT.	+

*CBCT* Cone beam computed tomography, *CT* Computed tomography, *DASH* Disability of the Arm, Shoulder and Hand, *FAC* Functional Ambulation Classification, *MHQ* Michigan Hand Outcomes Questionnaire, *MAYO* Modified Green O’Brien, *ND* Not described, *NRS* Numerical Rating Scale, *PRWE* Patient-Rated Wrist Evaluation, *VAS* Visual Analogue Scale

**Fig. 2:**
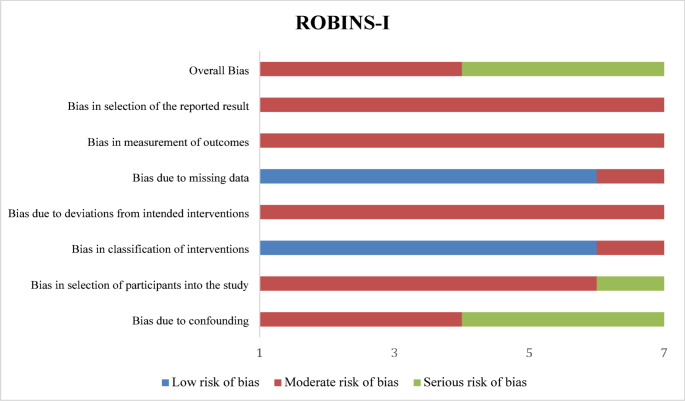
Risk of bias assessment for the non-randomised intervention studies (ROBINS-I)

Table 5; Fig. 2 summarize the risk of bias assessment of the included studies using the ROBINS-I tool. As shown in both the tabular and graphical presentations, most studies were judged to have an overall moderate risk of bias, primarily driven by confounding and participant selection. The graphical distribution (Fig. 2) illustrates that classification of interventions consistently showed low risk of bias, whereas moderate risk predominated in domains related to deviations from intended interventions, outcome measurement, and selective reporting. Overall, the majority of the included studies were classified as having a moderate overall risk of bias according to the ROBINS-I assessment. The quality of evidence for extracorporeal shockwave therapy in bone fractures, according to the GRADE approach, is presented in Table 6. The overall quality of evidence was rated as low for the outcomes fracture union rate and time to fracture union, reflecting limitations related to the non-randomized design of the included studies and the presence of inconsistency, indirectness, and imprecision. Despite these limitations, the findings suggest a potential beneficial effect of extracorporeal shockwave therapy on fracture healing outcomes.

**Table 5:** Risk of bias assessment of included studies using ROBINS-I

Study	Bias due to confounding	Bias in selection of participants into the study	Bias in classification of interventions	Bias due to deviations from intended interventions	Bias due to missing data	Bias in measurement of outcomes	Bias in selection of the reported result	Overall bias
Ahmed et al. 2022 [20]	Moderate	Moderate	Low	Moderate	Low	Moderate	Moderate	Moderate
Gökalp et al. 2023 [21]	Serious	Serious	Moderate	Moderate	Low	Moderate	Moderate	Serious
Mittermayr et al. 2022 [11]	Moderate	Moderate	Low	Moderate	Low	Moderate	Moderate	Moderate
Quadlbauer et al. 2019 [22]	Serious	Moderate	Low	Modetate	Moderate	Moderate	Moderate	Serious
Ramon et al. 2023 [23]	Moderate	Moderate	Low	Low	Low	Moderate	Moderate	Moderate
Wang et al. 2007 [24]	Serious	Moderate	Low	Moderate	Low	Low	Moderate	Serious
Yang et al. 2025[25]	Moderate	Moderate	Low	Moderte	Low	Moderate	Moderate	Moderate

*Low*  low risk of bias, *Moderate* moderate risk of bias, *Serious* serious risk of bias, *Critical* critical risk of bias

 .

**Table 6:** Summary of findings (GRADE) for extracorporeal shockwave therapy in bone fractures

Outcome	Number of studies	Study design	Limitations	Inconsistency	Indirectness	Imprecision	Publication bias	GRADE level of evidence
Fracture union rate	7	Non-randomized	Serious	Moderate	Moderate	Moderate	Undetected	⊕⊕◯◯ Low
Time to fracture union	5	Non-randomized	Serious	Moderate	Moderate	Moderate	Undetected	⊕⊕◯◯ Low

## Discussion

In the present study, the available literature about the effects of ESWT on the process of bone healing in fractures was analyzed. ESWT has been recently used for stimulating bone metabolism and non-consolidated fractures. However, there is a lack of established protocols for using ESWT for these proposal as can be seen in the studies used in this systematic review. The studies included female and male patients (ranging from 18 to 68 patients) with fractures at different sites such as mandibula, tibia and femur, scaphoid nonunion and clavicula. Also, many different parameters were used for the different authors with the energy flux density ranging from 0.21 mJ/mm² to 0.62 mJ/mm², pressure from 2.0 to 3.0 bar and pulses from 2000 to 6000. Moreover, many different sites of fractures were studied for the different authors such as scaphoid nonunion site. Also, the analysis used by the authors demonstrated that, after the treatment, stimulatory effects of ESWT were seen on the process of bone healing was seen. Moreover, functional and pain level assessments also demonstrated that positive effects after the treatment.

Analyzing the data of the papers, it was possible to observe a considerable heterogeneity in geographic origin, study design, and patient characteristics of the patients included in the present study, reflecting the growing international interest in the clinical application of ESWT for fracture healing. Despite methodological variability, most studies addressed fractures characterized by impaired biological healing, particularly delayed union and nonunion. Moreover, it is worthwhile to point out that the studies used a small sample size, indicating that the available evidence remains limited and largely exploratory. Nevertheless, the inclusion of prospective and randomized clinical studies demonstrates an increasing effort to strengthen the methodological rigor in this field [9]. Also, it is important to emphasize that the studies included a wide diversity of fracture types and anatomical locations, with most of the investigations focusing on fractures of long bones such as the tibia, femur, clavicle, and fibula. Those are frequently associated with mechanical instability and reduced vascular supply, factors known to contribute to delayed consolidation or nonunion [25, 26]. However, the inclusion of fractures in smaller bones, such as the scaphoid and metatarsals, suggests that the biological effects of ESWT may extend across different skeletal sites and fracture patterns [22, 23].

Although the use of ESWT for treating fractures is increasing in the clinical setting, there is no established protocol for a therapeutical intervention. This statement can be confirmed by the marked variability in treatment parameters, particularly regarding energy flux density, number of impulses, and treatment frequency. Energy levels ranged from 0.21 mJ/mm² to 0.62 mJ/mm², with most studies adopting intermediate intensities between approximately 0.35 and 0.41 mJ/mm². These values are consistent with those commonly described in the orthopedic literature as biologically effective for stimulating bone regeneration without inducing excessive tissue damage [27]. Previous experimental and clinical studies suggest that ESWT within this moderate energy range is able of triggering mechanotransduction processes in bone tissue, stimulating the expression of many different osteogenic and angiogenic mediators, which are essential for the recruitment of osteoprogenitor cells and the formation of new bone matrix [28, 29]. In contrast, it seems that higher energy levels (such as those used by Wang et al. [24] and Gökalp et al. [21], may produce stronger mechanical stimulation into bone tissue metabolism, potentially reactivating the healing cascade in biologically inactive fracture sites such as nonunions. Similarly, considerable heterogeneity was observed in the number of pulses and treatment sessions. Some studies applied 3000 pulses in a single treatment session and others applied lower pulse numbers delivered across multiple sessions or prolonged treatment regimens [9, 15, 25]. These variations may reflect attempts to modulate the cumulative biological stimulus delivered to the fracture site. Repeated mechanical stimulation could theoretically sustain cellular activation and enhance angiogenesis and osteogenesis over time, particularly in chronic fracture conditions characterized by impaired vascularization and reduced cellular activity. Furthermore, the authors used imaging guidance techniques (radiography, fluoroscopy, or ultrasonography) to localize the fracture focus during ESWT application. This approach is clinically relevant, as the biological effects of shockwaves are highly dependent on precise energy delivery to the pathological site. In this context, taking all the information together, it can be state that the therapeutic effect of ESWT is determined by a complex interaction between energy dose, number of impulses, and the biological status of the fracture environment [14, 30]. Moreover, different analysis was used to evaluate the effects of ESWT such as computed tomography, conventional radiographs and bone density. Also, functional evaluation was also used.

Despite the methodological differences, most of the studies reported positive outcomes associated with ESWT, progressive bone healing, and increase in bone mass and also improvement in pain levels and recovery of function or mobility. It is well known that the beneficial effects of extracorporeal shockwave therapy (ESWT) on bone healing are mediated by the mechanical stimulus from the shock waves on the cellular, and molecular levels, stimulating the biological cascade of fracture repair [31]. Some authors state that the acoustic pulses generated by ESWT produce controlled microtrauma and mechanical stress within the bone and surrounding tissues, activating mechanotransduction pathways that induce the release of growth factors associated with osteogenesis and angiogenesis [28, 32, 33]. It has been stated that all of the modifications produced by ESWT increases the expression of osteogenic markers such as bone morphogenetic proteins (BMP-2 and BMP-7), transforming growth factor-β1 (TGF-β1), and vascular endothelial growth factor (VEGF), all of which play critical roles in regulating bone formation and vascularization during fracture repair [34]. In addition, shockwave stimulation has been reported to enhance the proliferation and differentiation of mesenchymal stem cells and osteoprogenitor cells, facilitating their maturation into osteoblasts and promoting new bone matrix deposition [14, 32]. In addition, it has been demonstrated that a stimulation of neovascularization and improvement of local microcirculation were observed after ESWT application. As a result, an increase in oxygen and nutrient delivery to the fracture site were seen [28]. Another mechanism of action attributed to the application of shock wave is the observed increase in nitric oxide production and activation of signaling pathways involved in tissue repair [29, 35]. All the biological effects may explain the favorable radiographic consolidation and functional improvements observed in several of the studies included in this review, particularly in cases of delayed union and nonunion where the intrinsic healing response is impaired.

This systematic review presents limitations that should be considered when interpreting the findings. First, the number of eligible clinical studies investigating ESWT for fracture healing remains relatively small, which restricts the robustness of the overall conclusions. In addition, the substantial heterogeneity among studies regarding ESWT treatment parameters, including energy flux density, number of pulses, treatment sessions, and application protocols make difficult the comparison among the studies. Variability was also observed in fracture types, anatomical locations, patient characteristics, and follow-up periods, which may influence the biological response to shockwave therapy and complicate direct comparisons between studies. Furthermore, different imaging modalities and outcome measures were used to assess bone healing, ranging from conventional radiographs to computed tomography and cone beam computed tomography, which may introduce methodological inconsistencies in the evaluation of fracture consolidation. Finally, the relatively small sample sizes reported in most studies reduce statistical power and limit the generalizability of the findings.

## Conclusion

The studies included in this review reported positive radiographic and functional outcomes following the application of ESWT in the treatment of fractures, including cases of delayed union and nonunion, when compared to other therapeutic approaches or conventional treatment. However, the results should be interpreted with caution, considering the methodological heterogeneity among studies and the limited number of high-quality clinical trials available. In this context, although the findings suggest favorable outcomes, well-designed randomized controlled trials are still needed to more consistently confirm the clinical effectiveness of ESWT.

## Data Availability

No datasets were generated or analysed during the current study.
